# Sodium Propionate Contributes to Tumor Cell Growth Inhibition through PPAR-γ Signaling

**DOI:** 10.3390/cancers15010217

**Published:** 2022-12-29

**Authors:** Alessia Filippone, Giovanna Casili, Sarah Adriana Scuderi, Deborah Mannino, Marika Lanza, Michela Campolo, Irene Paterniti, Anna Paola Capra, Cristina Colarossi, Annalisa Bonasera, Sofia Paola Lombardo, Salvatore Cuzzocrea, Emanuela Esposito

**Affiliations:** 1 Department of Chemical, Biological, Pharmaceutical and Environmental Sciences, University of Messina, Viale Ferdinando Stagno D׳Alcontres, 31-98166 Messina, Italy; 2 Istituto Oncologico del Mediterraneo, Via Penninazzo, 7-95029 Catania, Italy

**Keywords:** apoptosis, autophagy, glioblastoma, PPAR-γ, short-chain fatty acids

## Abstract

**Simple Summary:**

Glioblastoma (GBM) is the most aggressive primary brain tumor with no positive outcome. Interesting pieces of evidence indicate the short-chain fatty acids (SFCAs) role in tumor growth attenuation. In fact, colon or breast cancers have been reported to be reduced by SCFA treatment. Remarkably, among several pathways frequently deregulated in GBM and driven by SCFAs like NF-kB, apoptosis and autophagy pathways result in SCFAs activity. Subsequently, peroxisome proliferator-activated receptor (PPAR) strongly correlates with the SCFAs activity in tumors. Therefore, strategies aimed at targeting sodium propionate (SP) belonging to SCFAs could be a promising approach in inducing apoptosis and autophagy pathways for GBM treatment through interaction with type γ of the PPAR receptor.

**Abstract:**

New therapeutic approaches are needed to improve the outcome of patients with glioblastoma (GBM). Propionate, a short-chain fatty acid (SCFA), has a potent antiproliferative effect on various tumor cell types. Peroxisome proliferator-activated receptor (PPAR) ligands possess anticancer properties. We aimed to investigate the PPAR-γ/SCFAs interaction in in vitro and in vivo models of GBM. The U87 cell line was used in the in vitro study and was treated with sodium propionate (SP). U87 cells were silenced by using PPAR-γ siRNA or Ctr siRNA. In the in vivo study, BALB/c nude mice were inoculated in the right flank with 3 × 10^6^ U-87 cells. SP (doses of 30 and 100 mg/kg) and GW9662 (1 mg/kg) were administered. In vitro exposure of GBM to SP resulted in prominent apoptosis activation while the autophagy pathway was promoted by SP treatments by influencing autophagy-related proteins. Knockdown of PPAR-γ sensitized GBM cells and blocked the SP effect. In vivo, SP was able to decrease tumor growth and to resolve GBM tissue features. SP promoted apoptosis and autophagy pathways and tumor cell proliferation leading to cell cycle arrest through a PPAR-γ-dependent mechanism suggesting that the PPAR-γ/SCFAs axis could be targeted for the management of GBM.

## 1. Introduction

Glioma is an umbrella term used for glial brain tumors including astrocytic tumors, oligodendroglial tumors, oligoastrocytoma tumors, and ependymal tumors. Glioblastoma (GBM) is a World Health Organization (WHO) grade IV brain tumor which includes the deadliest worldwide human diseases. GBM rises proportionally with age and male gender showing the most marked frequency in 70–85-year-old individuals [1]. Tumorigenesis in malignant tumors such as GBM has been associated with an instability of multiple cellular metabolic processes including apoptosis, angiogenesis, and DNA repair mechanisms [2]. These well-established processes are comprised of certain factors and receptors considered main players of recognized cellular degradative processes such as apoptosis and autophagy: the “genome guardian” p53 protein and the “autophagosome founder” microtubule-associated protein 1 light chain (LC3). First, GBM cells are resilient to going through programmed cell death and instead activates signal transduction pathways and a various number of negative/positive regulators after the p53 response [3]. Indeed, the p53 pathway negatively influences the production of the Bcl-2 family and inhibitor of apoptosis (IAP) proteins and drives the production of genes encoding for Bax, caspase-3, cyclin-CDK, and p38 MAP kinase proteins [4]. More specifically, the positive feedback loop of the expression levels of these proteins is altered in GBM cells suggesting that the induction of apoptosis seems to be considered an encouraging approach for counteracting GBM progression. Second, the LC3 player is considered an activator of the autophagy pathway, and together with autophagy-related protein (Atgs), is involved in the formation of the autophagosome that can transport materials to the lysosome for degradation. The autophagy pathway digests unwanted proteins and organelles to obtain energy and sustain basal cell turnover and represents a possible therapeutic target for neurodegenerative diseases including Alzheimer’s disease [5,6] and Parkinson’s disease [7] through clearance of amyloid beta (A-β) and a-synuclein (α-syn) aggregates, respectively. In addition, for GBM management, autophagy activation can result in tumor suppression, proliferation impediment, and attenuated invasion in both human GBM and glioma cells [8,9,10].

Short-chain fatty acids (SCFAs) such as acetate, propionate, and butyrate are considered the main energy source for intestinal mucosa cells, particularly colonocytes because SCFA production occurs thanks to microbiota flora after fiber and complex carbohydrates ingestion. SCFAs have been reported to possess multiple protective effects including modulation of the inflammatory cascade through inhibition of the NF-κB and histone deacetylase pathways. In addition, sodium butyrate (SB) and sodium propionate (SP), sodium salts of butyric acid and propionic acid, respectively, were able to modulate the immune system, oxidative stress, interleukins (ILs)/cytokines release, and inflammation of the central nervous system in many in vitro and in vivo studies [11,12,13]. Recently, SCFAs have been proposed as anti-cancer agents for colon and breast cancer by promoting apoptosis and cell detachment, and also counteracting tumor cell number within the tumor microenvironment (TME). In addition, it has been reported that there is a strong connection between SCFAs and peroxisome proliferator-activated receptors (PPARs) by stimulation of different routes of signaling including angiopoietin, lipogenesis pathways, and gut dysbiosis in adenocarcinoma, colorectal, and breast cancer cells [14,15,16].

Peroxisome proliferator-activated receptor gamma (PPAR-γ), a member of the nuclear receptor family of ligand-activated transcription factors regulates gene expression of multiple signaling pathways including B-cell lymphoma 2 (BCL-2), nuclear factor kappa-light-chain-enhancer of activated B cells (NF-κB), tumor suppressor p53 (p53), and cyclooxygenase-2 (COX-2) mediators that can regulate different diseases including cancers [17]. In the cancer context, PPAR-γ can negatively regulate the cell cycle, proliferation, and mobility of bladder cancer cells, and PPAR-γ agonists can reduce cell viability of GBM in different cell lines. Although PPAR-γ has been consistently observed to be expressed in various cancer cell lines, its function in tumorigenesis still remains controversial and needs further elucidation. Moreover, the interaction between SP and PPAR-γ should be defined since it has been proposed that sodium propionate targets PPAR-γ indirectly via signaling intermediates such as the SCFA receptor GPR43 (https://doi.org/10.3389/fendo.2014.00085, accessed on 25 November 2022), HDAC (https://doi.org/10.1038/s41401-020-0402-x, accessed on 25 November 2022), or the Wnt/β-catenin pathway (https://doi.org/10.1186/1756-0500-7-226, accessed on 25 November 2022). Moreover, SCFAs production after fiber fermentation and enterocyte activity induces a highly mucosal response that may be governed by PPAR-γ (https://doi.org/10.1002/mnfr.201400597, accessed on 25 November 2022).

Therefore, the main objectives of this study were: (i) to explore the hypothesis that apoptosis and autophagy pathways can be modulated by one of the most studied SCFAs, propionate and (ii) to elucidate whether and how PPAR-γ is involved in these changes by affecting tumor progression in GBM cells and an in vivo xenograft model.

## 2. Materials and Methods

### 2.1. Materials

SP (Cat #P1880), GW9662 (MedChemExpress, Cat #HY16578), and all other chemicals were obtained from the Sigma–Aldrich Company (Milan, Italy). SP was dissolved in DMEM (in vitro study) or saline (in vivo study). GW9662 was dissolved in DMSO (≥100 mg/mL, in vitro study; 10%, in in vivo study) following the manufacturer’s instructions.

### 2.2. In Vitro Study

#### 2.2.1. Cell Culture

The normal human astrocyte cell line NHA (NHA-Astrocytes AGM LONZA^®^ CC-2565™ Homo sapiens astrocytes) and the human GBM cell lines U-87 (U-87 MG ATCC^®^ HTB-14^TM^ Homo sapiens brain glioblastoma IV grade), U-138MG (U-138 MG ATCC^®^ HTB-16™ Homo sapiens brain glioblastoma IV grade), and A-172 (A-172 ATCC^®^ CRL-1620™ Homo sapiens brain glioblastoma) were used in this study and purchased from the ATCC (American Type Culture Collection, Rockville, MD, USA). The NHA cell line was cultured in 25 cm^2^ flasks with the CC-3186 AGM™ BulletKit™ [which contains a 500 mL bottle of ABM™ (CC-3187) and AGM™ SingleQuots™ (CC-4123)] at 37 °C in 5% CO_2_. GMB human cells were cultured in 75 cm^2^ flasks with Dulbecco’s modified Eagle’s medium (DMEM—Sigma-Aldrich^®^ Catalog No. D5030; St. Louis, MO, USA) complemented with antibiotics (penicillin 1000 units—streptomycin 0.1 mg/L, Sigma-Aldrich^®^ Catalog No. P4333; St. Louis, MO, USA), L-glutamine (GlutaMAX^TM^, ThermoFisher Scientific^®^ Catalog No. 35050061; Waltham, MA, USA), and 10% (*v*/*v*) fetal bovine serum (FBS, Sigma-Aldrich^®^ Catalog No. 12103C; St. Louis, MO, USA) in a humidified atmosphere of 5% CO_2_ and maintained at 37 °C.

#### 2.2.2. Cell Treatments

**Scheme 1:**
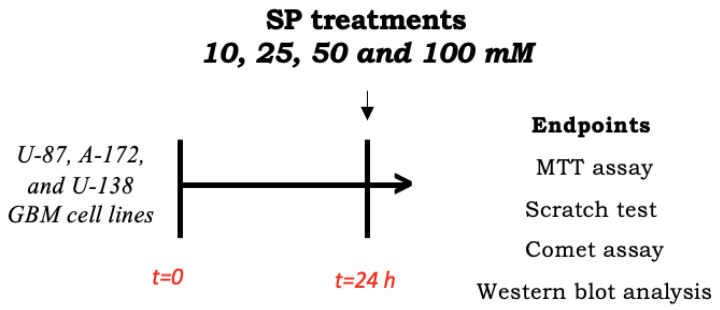
Study design of in vitro experiment. To investigate SP effects in inducing apoptosis and autophagy pathways, U87 cells were seeded and after 24 h, treated with SP at different concentrations. After 24 h, cell lysates were collected and used for analyses.

Human GBM cells (U-87, A-172, and U-138 GBM cell lines) were plated on 96-well plates at a density of 4 × 10^4^ cells/well to a final volume of 150 μL. After 24 h, GBM cells were treated for 24 h with SP at concentrations of 10 mM, 25 mM, 50 mM, or 100 mM dissolved in the basal medium (Scheme 1).

#### 2.2.3. Cell Viability Assay (MTT Assay)

Cell viability assays on GBM cells were performed using the 3-(4,5-dimethylthiazol-2l)-2,5-diphenyltetrazolium bromide assay (MTT assay). U-87, A-172, and U-138 GBM cells were plated on 96-well plates (Corning Cell Culture, Corning, NY, USA) at a density of 4 × 10^4^ cells/well to a final volume of 150 μL and after 24 h they were treated with different concentrations of SP (10, 25, 50, or 100 mM) (Scheme 1). After 24 h, the cells were incubated at 37 °C with MTT (0.2 mg/mL) for 1 h. Then, the medium was removed and cells were lysed with 100 μL dimethyl sulfoxide (DMSO). The amount of MTT reduced to formazan was quantified by measuring the optical density (OD) at 550 nm with a microplate reader.

#### 2.2.4. Wound Healing Assay (Scratch Test)

The scratch assay was performed to study the effects of SP on U-87 GBM cell migration according to [18]. Briefly, U-87 cells around 80% confluency were detached and 2 × 10^6^ U-87 GBM cells were plated on 60 mm plates (Corning Cell Culture, Tewksbury, MA, USA) in a volume of 2 mL. After 24 h, the cell layer was scratched with a p200 pipette tip to create a straight line. Subsequently, debris was removed from each plate and replaced with normal culture medium in the control group and treated for 24 h with SP at different concentrations (10, 25, 50, or 100 mM) (Scheme 1). Finally, each plate was placed under a microscope and photographed. Images of the cells were captured and the wound width at the marked wound locations after 24 h was used to measure the migratory ability of the cells. The cell migration rate was analyzed and calculated using Image J (1.53a version) software.

#### 2.2.5. Alkaline (pH > 13) Comet Assay

Alkaline microgel electrophoresis or comet assay was performed to detect DNA strand breaks in individual cells. The principle of the test is that under an electric field, the fragmented DNA migrates out of the nucleoid body (known as “comet head”) and forms a DNA stain in the agarose gel (defined as the “comet tail”). The comet assay was performed using U-87 GBM cells as previously described [19]. Briefly, a 20 μL aliquot of cells (2 × 10^3^ cells/μL) was added to 180 μL of agarose (0.7% low-melting agarose). After mixing, the samples were pipetted onto an area of the comet slide. The slide was incubated at 4 °C for 15 min and then transferred to pre-chilled lysis solution containing 1.5 M NH_4_Cl, 10 mM EDTA, and 100 mM NaHCO_3_ for 30 min at 4 °C. Denaturation was performed in an alkaline solution containing 0.3 M NaOH and 1 mM EDTA at RT for 30 min. The slides were then moved to TBE containing 300 mM NaOH and 1 mM EDTA for 10 min. Electrophoresis was conducted in a horizontal chamber (25 V, 0.96 V/cm, 300 mA) for 15 min. The slide was then fixed in ice-cold, 100% ethanol for 5 min, air-dried, and stained with 30 μL of 20 μg/mL ethidium bromide (Scheme 1). The percentage of DNA in the comet’s tail (% TDNA) was used as a DNA damage parameter. The results were reported as the comet’s tail length obtained from fluorescence (% tail intensity), which is an indication of the presence of DNA damage.

#### 2.2.6. Cell Transfection

**Scheme 2:**
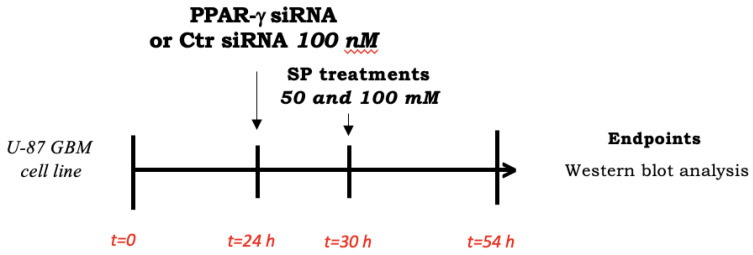
Study design of in vitro silencing of PPAR-γ. To investigate SP effects in inducing apoptosis and autophagy pathways through PPAR-γ signaling, U87 cells were seeded and after 24 h, stimulated with PPAR-γ or Ctr siRNA for 6 h. Then, fresh basal medium contained SP at different concentrations was added. After 24 h, cell lysates were collected and used for analyses.

U-87 GBM cells at ∼70% confluency were seeded on a 6-well plate and transfected after 24 h with 100 nM siRNA against PPAR-γ (#AM16708, siRNA ID 143093, Invitrogen by Thermo Fischer Scientific, Waltham, MA USA) or 100 nM control siRNA (#AM4611, Invitrogen by Thermo Fischer Scientific, Waltham, MA USA) for 6 h using Lipofectamine transfection reagent (#18324-020, Invitrogen by Thermo Fischer Scientific, Waltham, MA USA) as previously described [5]. Briefly, two mixes were prepared: a mix of normal culture medium (DMEM—Sigma-Aldrich^®^ Catalog No. D5030; St. Louis, MO, USA) with 100 nM control siRNA and a mix of normal culture medium (DMEM—Sigma-Aldrich^®^ Catalog No. D5030; St. Louis, MO, USA) with 100 nM PPAR-γ siRNA; both mixtures were incubated for 5 min at room temperature. Next, the prepared solutions were mixed with the Lipofectamine transfection reagent and incubated for 15 min according to the manufacturer’s instructions. The mixture was added to each well (250 μL/well) that already containing 1 mL of DMEM. After 6 h of incubation, the cells were treated for 24 h with SP at a concentration of 50 or 100 mM while the normal culture medium was added to the siRNA control group and PPAR-γ siRNA (Scheme 2).

#### 2.2.7. Western Blot Analysis

Western blot analysis using U-87 GBM cell lysates was performed as previously described by [20]. Human GBM cells were washed twice with ice-cold phosphate-buffered saline (PBS), collected, and resuspended in 20 mM Tris-HCl pH 7.5, 10 mM NaF, 150 μL NaCl, 1% Nonidet P-40, and protease inhibitor cocktail (Roche, Monza, Italy). After 40 min, the cell lysates were centrifuged at 16,000× *g* for 15 min at 4 °C. Protein concentration was estimated by the Bio-Rad protein assay using bovine serum albumin as the standard. The samples were then heated to 95 °C for 5 min and equal amounts of proteins were separated on a 10–15% SDS-PAGE gel and transferred to a PVDF (Immobilon-P) membrane. The membranes were incubated overnight at 4 °C with the following primary antibodies: anti-p53 (1: 500; Santa Cruz Biotechnology, Dallas, TX, USA; sc-126), anti-caspase 3 (1:500; Santa Cruz Biotechnology, sc 7272), anti-Bax (1: 500; Santa Cruz Biotechnology, Dallas, TX, USA; sc-7480), anti-Bcl2 (1: 500; Santa Cruz Biotechnology, Dallas, TX, USA; sc-7382), anti-MAPLC3 or LC3 (1:1000 Santa Cruz Biotechnology, sc-271625), anti-p-62 (1:1000, Abcam, ab91526), anti-Beclin-1 (1:500 Santa Cruz Biotechnology, sc-48341), anti-Atg 5 (1:1000; Santa Cruz Biotechnology, sc-133158), anti-PPAR-γ (1:500, Santa Cruz Biotechnology sc-7273), anti-PPAR-α (1:500, Santa Cruz Biotechnology,sc-398394), anti-PARP1 (1:500, Santa Cruz Biotechnology, sc-8007), or anti-PPAR-β (1:500, Santa Cruz Biotechnology, sc-74517). The stripping and re-probing of the WB membrane were performed for detecting some antibodies. To ensure that the blots were loaded with equal amounts of protein lysate, they were also incubated with the β-actin antibody (1: 500; Santa Cruz Biotechnology; Dallas, TX, USA. Sc-8432). The signals were detected with the Advanced Chemiluminescence Detection System (ECL) reagent according to the manufacturer’s instructions (Thermo Fisher, Waltham, MA, USA). The relative expression of the protein bands was quantified by densitometry with the BIORAD ChemiDoc^TM^XRS+ software and standardized to β-actin levels as an internal control.

### 2.3. In Vivo Study

#### 2.3.1. Animals

BALB/c nude male mice (25–30 g; 6–8 weeks of age) were purchased from Envigo (Milan, Italy). Animals were placed in a controlled environment and were fed with a standard diet and water ad libitum under pathogen-free conditions with a 12 h light/12 h dark cycle. The animal study was approved by the University of Messina (n° 783/2021-PR) following Italian regulations on the use of animals (D.M.116192) and Directive legislation (EU) (2010/63/EU) amended by Regulation (EU) 2019/1010.

#### 2.3.2. Xenograft Model of GBM

**Scheme 3:**
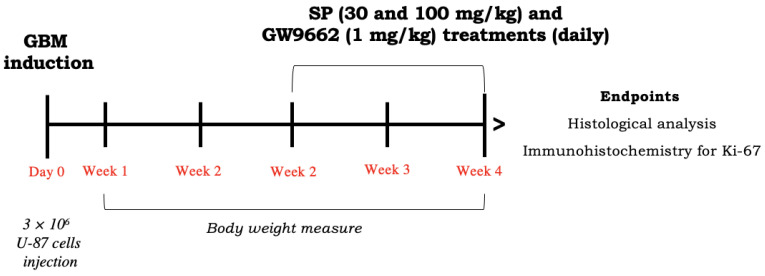
Study design of in vivo experiment. To investigate SP effect in counteracting GBM progression through PPAr-γ signaling, mice were inoculated with U87 cells (day 0). After 2 weeks, SP was administered for 2 weeks at the doses of 30 and 100 mg/kg in combination with GW9662 (1 mg/kg). After 4 weeks, GBM mass was collected and used for analyses.

Mice were subcutaneously injected into the right flank with 3 × 10^6^ U-87 GBM cells in 0.2 mL of PBS and 0.1 mL of Matrigel (BD Bioscience, Bedford, MA, USA) [21]. After tumor cell inoculation, animals were monitored daily for morbidity and mortality, and their body weight was monitored weekly to evaluate overall health. When tumor size reached about 200–300 mm^3^, about two weeks after GBM induction, mice were orally treated with SP. Measurement of tumor size could be performed in vivo during the experiment due to the subcutaneous localization of the tumor and it was performed with a digital caliper. SP was orally administered at a dose of 30 mg/kg or 100 mg/kg dissolved in saline for two weeks. The doses of SP were established based on previous studies [11,12,13,22]. The selective PPAR-γ antagonist GW9662 (Sigma-Aldrich, Milan, Italy) was administered by intraperitoneal injection (i.p.) at a dose of 1 mg/kg and was dissolved in a 1:1:8 mixture of ethanol: Tween80: saline; the dose was chosen according to its IC50 and based on previous studies [23]. The mice were randomly divided into seven experimental groups, as described below (Scheme 3):

1. Control group (GBM): oral administration of saline;
2. GBM + SP 10 mg/kg: after cell inoculation, SP at a dose of 10 mg/kg was orally administered for two weeks (n = 10);
3. GBM + SP 30 mg/kg: after cell inoculation, SP at a dose of 30 mg/kg was orally administered for two weeks (n = 10);
4. GBM + SP 100 mg/kg: after cell inoculation, SP at a dose of 100 mg/kg was orally administered for two weeks (n = 10);
5. GBM + SP 10 mg/kg + GW9662 1 mg/kg: after cell inoculation, SP (10 mg/kg) was orally administered and GW9662 (1 mg/kg) was administered intraperitoneally for two weeks (n = 10);
6. GBM + SP 30 mg/kg + GW9662 1 mg/kg: after cell inoculation, SP (30 mg/kg) was orally administered and GW9662 (1 mg/kg) was administered intraperitoneally for two weeks (n = 10);
7. GBM + SP 100 mg/kg + GW9662 1 mg/kg: after cell inoculation, SP (100 mg/kg) was orally administered and GW9662 (1 mg/kg) was administered intraperitoneally for two weeks (n = 10).

Mice were sacrificed four weeks after U-87 GBM cell injection and the tumor mass was excised for analysis and measured using a digital caliper and calculated using the equation V = W^2^ × L/2, where W and L represent minor and major lengths.

#### 2.3.3. Histological Evaluation

Histological analysis was performed as previously described [24]. For histopathological analysis, we have shown xenograft GBM tumors that consisted of immortalized GBM cells in rapid proliferation that originated from the solid tumor mass in the right flank of the mice. The collected tumor mass tissues of each experimental group were fixed in 10% (*w*/*v*) PBS-buffered formaldehyde solution at 25 °C for 24 h, dehydrated with graduated ethanol, and embedded in paraffin to obtain 7 μm thick sections. Hematoxylin and eosin (H&E) staining was performed on previously deparaffinized sections to study histological changes. The stained sections were examined by light microscopy (AxioVision, Zeiss, Milan, Italy). Tumor samples were evaluated by using a 0–5-point scale that referred to several parameters such as marked hypercellularity, nuclear atypia, microvascular proliferation, and necrosis (0 = no tumor spreading, 1 = poor tumor differentiation, 2 = moderate differentiation and nuclear atypia, 3 = differentiated tumor cells, 4 = well defined nuclear atypia and spreading, 5 = extreme spreading). Representative images were taken at 20× magnification (50 µm scale bar) and 40× magnification (20 µm scale bar).

#### 2.3.4. Enzyme-Linked Immunosorbent Assay (ELISA) Kits

ELISA kits were used to detect the levels of Ki-67 (Cat. No MBS1601117) [20], caspase-3 (Cat. No MBS849298-100), BAX (Cat. No ab233624), Bcl2 (Cat. No MBS2881897), p62 (Cat. No MBS3806181), LC3II (Cat. No LS-F55215), and Atg5 (Cat. No MBS2104990) in tumor mass tissues of mice according to the manufacturer’s protocols.

### 2.4. Statistical Analysis

All values are given as mean ± standard deviation (SD) of “n” observations. The results were analyzed by one-way analysis of variance (ANOVA) followed by a Bonferroni post-hoc test for multiple comparisons. A *p*-value of less than 0.05 was considered significant.

## 3. Results

### 3.1. In Vitro Study

#### 3.1.1. SP Treatments Reduced GBM Cell Viability in a Concentration-Dependent Manner

**Figure 1:**
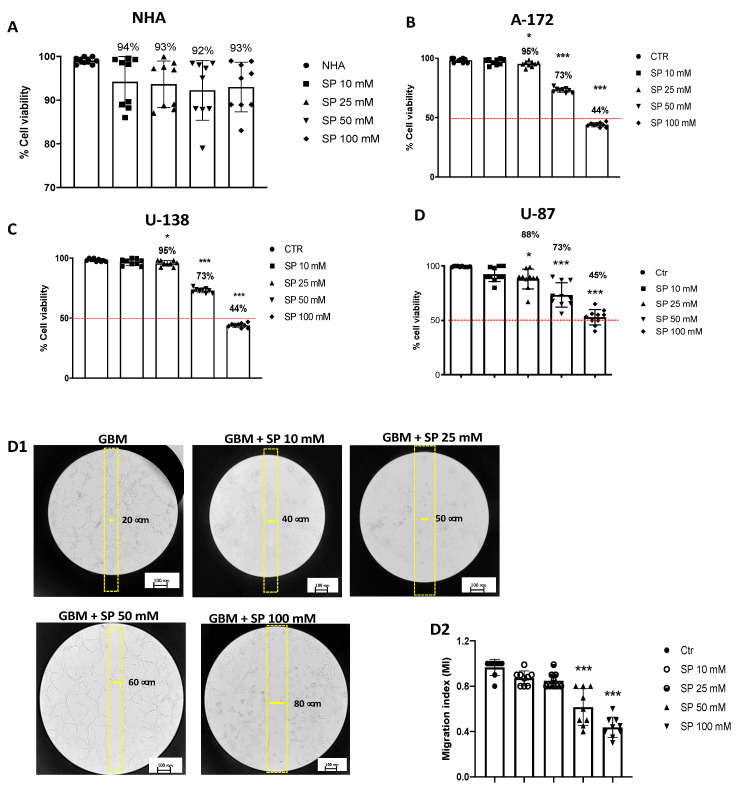
SP treatments reduce viability and inhibit migration of GBM cells. (**A**) MTT assay demonstrated that SP treatments for 24 h at the concentrations of 10, 25, 50, and 100 mM have no cytotoxic effect in healthy NHA cells. (**B**–**D**) Cell viability of U-87, A-172, and U-138 GBM cells was evaluated using MTT assay 24 h after treatments with SP at a concentration of 10, 25, 50, or 100 mM s. U-87, U-138, and A-172 cells showed a similar decrease in cell viability following SP treatments. Treatment with 10 mM SP did not significantly reduce cell viability, while concentrations of 25 mM, 50 mM, and 100 mM significantly reduced cell viability compared to untreated cells. * *p* < 0.05 vs. Ctr; *** *p* < 0.001 vs. Ctr. (**D1**,**D2**) U-87 cells were examined for cell invasion in 60 mm plates by carrying out the Scratch test. (**D1**) The wound was photographed 24 h after SP treatments to measure the migratory ability of the cells. (**D2**) SP treatments at the concentrations of 10 mM and 25 mM did not significantly affect cell migration rate, while invasiveness of U-87 cells was significantly reduced by SP treatment at the higher concentrations of 50 mM and 100 mM compared to untreated cells. *** *p* < 0.001 vs. Ctr. Red line = 50 % of cellular viability.

We also evaluated the cytotoxicity of SP in NHA cells as a control [25,26,27]. The cell viability assay demonstrated that all SP concentrations (10, 25, 50, and 100 mM) have no cytotoxic effect in healthy NHA cells (Figure 1A). U-87, A-172, and U-138 GBM cells were treated with different concentrations (10, 25, 50, and 100 mM) of SP for 24 h to determine if high concentrations cause higher toxicity for these cells. It was observed a decrease in GBM cell viability in a concentration-dependent manner (Figure 1B–D). The viability of SP-treated cells at the lower concentration of 10 mM was not significantly changed while the higher concentrations showed a decrease in cell viability suggesting that SP was able to inhibit GBM cell proliferation (Figure 1B–D). Since the concentrations of 50 and 100 mM of SP produced the most potent cytotoxic effects, we decided to use them for further analysis. Additionally, SP showed similar effects on cell viability in all GBM cell cultures (Figure 1D); consequently, we decided to study the effect of SP on the U-87 cell line only. The U-87 cell line was chosen because it represents one of the most validated cell lines in the field of GBM research. In particular, the U-87 cell genome was recently sequenced showing that genetic aberrations from the original tumors were maintained in this cell line allowing a detailed study of the signaling pathways of oncogenic cells [28,29].

#### 3.1.2. SP Treatments Inhibited GBM Cell Migration

Cell migration is an essential step towards increasing the invasive potential of tumor cells and consequently cellular metastasis [30]. We found that SP treatments at the lower concentrations of 10 and 25 mM did not significantly affect cell migration rate since the number of cells that migrated into the scratch area were similar to the untreated cells. Meanwhile treatments with SP at the higher concentrations of 50 and 100 mM significantly reduced the migration of U-87 GBM cells (Figure 1D1,D2). According to these results, we reported the ability of SP to inhibit the migration of GBM cells.

#### 3.1.3. Apoptosis and Autophagy Pathways Are Induced by SP Treatments

**Figure 2:**
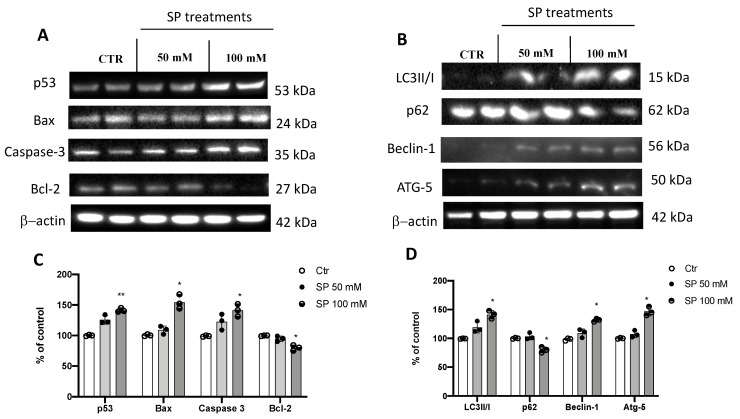
SP treatments induce apoptosis and autophagy pathways in GBM cells. (**A**) Western blot analysis of U-87 cell lysates revealed an increased expression of p53, Bax, and caspase-3 following SP treatments at concentrations of 50 and 100 mM in a dose-dependent manner. SP at the concentrations of 50 mM and 100 mM reduced Bcl2 expression compared to Ctr cells in a dose-dependent manner. (**C**) Densitometric analysis of immunoreactivity to antibodies shown in panel A. (**B**) Blots of U-87 cell lysates revealed an increase in LC3II/I, Beclin-1, and Atg-5 levels after 50 mM and 100 mM SP treatments compared to Ctr cells in a dose-dependent manner. p62 expression levels were significantly reduced after SP treatment at the higher concentration of 100 mM compared to Ctr cells. (**D**) Densitometric analysis of immunoreactivity to antibodies shown in panel (**B**). The data are representative of at least three independent experiments. ** *p* < 0.01 vs. Ctr; * *p* < 0.05 vs. Ctr.

The apoptosis process is compromised in GBM leading to a survival advantage of tumor cells and thus promoting tumor progression [31]. In GBM, the expression levels of the pro-apoptotic protein p53 was found to be low while cells treated with SP at the higher concentration of 100 mM increased the expression of p53. In addition, a significant increase in caspase-3 expression levels was observed in cells treated with SP at 50 and 100 mM compared to the untreated cells. BAX, another pro-apoptotic protein, showed an increase in its expression levels in SP-treated cells at the concentration of 100 mM. Conversely, the expression levels of the anti-apoptotic protein Bcl-2 was significantly reduced in the SP 100 mM-treated cells compared to the untreated cells, while the SP 50 mM treatment group did not show a significant reduction (Figure 2A,C). In addition to the apoptosis pathway, induction of the autophagic process causes cell death by lysosomal hyperactivation and represents a therapeutic target for the treatment of GBM [32]. Here, the expression levels of LC3, the main protein responsible for the formation of the autophagosome, were found to be significantly increased by SP treatments at the concentration of 100 mM [33], compared to the untreated GBM cells. In addition, an increase in expression levels of Beclin-1, a pro-autophagic protein involved in the formation of the Vps34–Vps15 complex [7], was observed in SP-treated cells at the concentration of 100 mM. We also evaluated the expression of the Atg5 protein, a key protein involved in the extension of the phagophore membrane in autophagic vesicles [34]; we found an increase in expression when cells were treated with SP at the concentration of 100 mM. Moreover, p62 degradation, an index of autophagic flow block [35], was evaluated. SP treatments decreased p62 expression levels when compared to untreated GBM cells, with a significant reduction at the higher concentration of SP (100 mM) (Figure 2B,D). Our results indicated that SP was able to regulate and influence autophagy and apoptosis by activating both pathways in GBM cells.

#### 3.1.4. SP Triggered DNA Strand Breaks in GBM Cells

**Figure 3:**
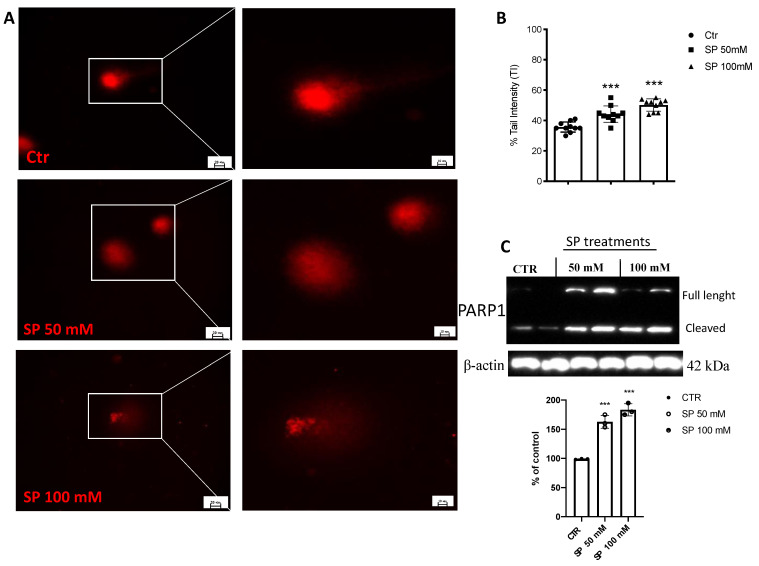
Effect of SP on DNA damage in GBM cells. (**A**) Representative microscopy images of the alkaline comet assay. Comet patterns were recorded after SP treatments at the concentrations of 50 and 100 mM and showed a significant increase in DNA damage compared to Ctr cells. (**B**) Densitometric analysis of comet assay. Microscopy images were observed and photographed at 40× and 100× magnification. (**C**) A baseline level of cleaved PARP was observed in cell lysates of the Ctr cells, while treatments with SP at concentrations of 50 and 100 mM significantly increased its expression. *** *p* < 0.001 vs. CTR.

Alterations in DNA damage/repair systems are cellular events that drive tumor initiation and progression [36]. Intact DNA was observed in untreated GBM cells. However, when U-87 cells were treated with SP, concentrations of 50 and 100mM breached DNA as visible by the increased length of the DNA comet tail (Figure 3A,B). Furthermore, to confirm that SP induced DNA damage in U-87 cells, we evaluated the expression of nuclear protein poly (ADP-ribose) polymerase 1 (PARP-1). As expected, we found that PARP was overexpressed by SP treatments at both concentrations of 50 and 100 mM when compared to the untreated cells (Figure 3C). The obtained results affirm that SP induces some damage to the DNA of the U-87 GBM cell leading to slowing of the cancer progression.

#### 3.1.5. Effect of SP on Peroxisome Proliferator-Activated Receptors (PPARs): PPAR-α, PPAR-β, and PPAR-γ Expression

**Figure 4:**
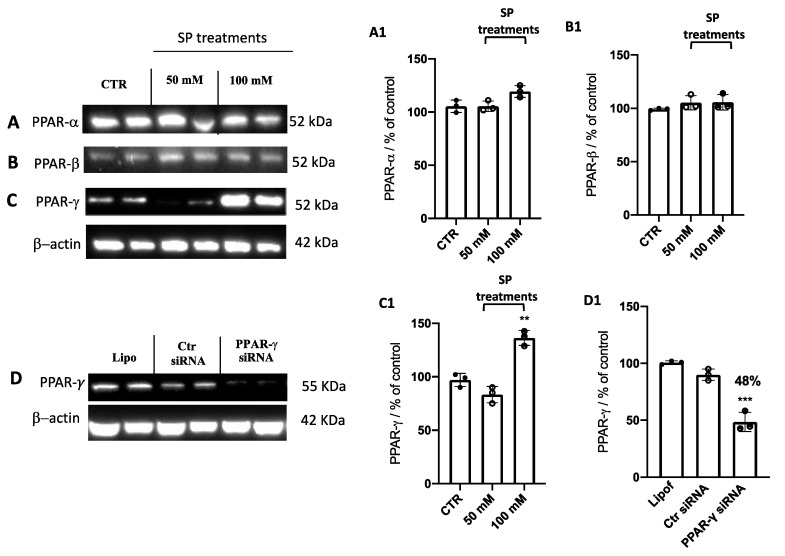
Effect of SP on PPAR expression levels. (**A**–**C**) Blots of U-87 cell lysates revealed that SP at the concentrations of 50 and 100 mM did not affect the expression of PPAR-α and PPAR-β compared to the Ctr cells. An increase in PPAR-γ expression was revealed after SP treatment at a higher concentration of 100 mM compared to the Ctr cells. (**A1**–**C1**) Densitometric analysis of the immunoreactivity to the antibody shown in panels (**A**–**C**). ** *p* < 0.01 vs. ctr. (**D**) Representative Western blot analysis for PPAR-γ in cell lysates transfected with 100 nM PPAR-γ siRNA, Ctr siRNA, or lipofectamine (Lipo) alone. A significant reduction of PPAR-γ expression was observed in PPAR-γ siRNA cells compared to control siRNA cells. (**D1**) Densitometric analysis of the immunoreactivity to the antibody shown in panel D. *** *p* <0.001 vs. Ctr siRNA.

Current studies have revealed different roles of PPARs in various types of cancers, including GBM [37,38,39,40,41]. As PPARs are activated by fatty acids and their derivatives creating a lipid signaling network between the cell surface and the nucleus [42,43], PPAR-α, PPAR-β, and PPAR-γ expression levels were evaluated to explore the possible molecular mechanism of SP acting through PPAR signaling. We found that the expression levels of PPAR-α (Figure 4A,A1) and PPAR-β (Figure 4B,B1) were not influenced by SP treatments. Despite no changes observed in PPAR-α and PPAR-β expression after SP treatments when U-87 GBM cells were treated for 24 h with SP at higher concentrations of 50 and 100 mM, we observed that the expression levels of PPAR-γ were increased compared to the untreated cells (Figure 4C,C1). Here, we reported that SP increased the expression of PPAR-γ but not PPAR-α or PPAR-β, and thus we hypothesized that SP could possess the ability to positively regulate PPAR-γ activity.

#### 3.1.6. SP Stimulated Apoptosis and Autophagy Pathways in GBM through PPAR-γ Signaling

**Figure 5:**
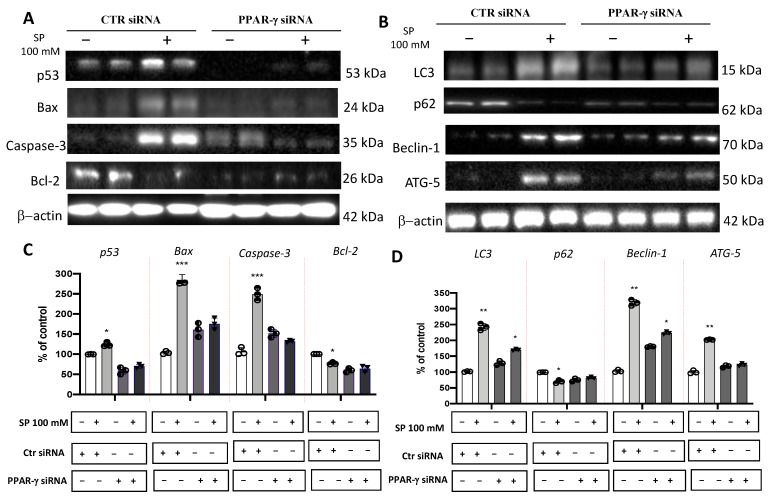
Evaluation of SP treatments on apoptosis and autophagy pathways following PPAR−γ short interfering RNA (siRNA) knockdown. (**A**) Representative western blots showed that p53, Bax, and caspase-3 expression levels were significantly increased in SP-treated cells at the higher concentration of 100 mM compared to the Ctr siRNA cells. Bcl−2 expression levels were significantly reduced in the SP−treated cells compared to the Ctr siRNA cells. No significant change in expression levels of p53, Bax, Caspase−3, and Bcl−2 was reported in Ctr siRNA and PPAR−γ−silenced cells also treated with SP. (**C**) Densitometric analysis of immunoreactivity to antibodies shown in panel (**A**). (**B**) Representative Western blots for LC3II/I, p62, Beclin−1, and Atg5. A significant increase in the expression levels of LC3II/I, Beclin−1, and Atg5 was observed in Ctr siRNA cells treated with SP at the concentration of 100 mM compared to Ctr siRNA untreated cells, while no significant increase was observed in PPAR−γ−silenced cells treated with SP at the concentration of 100 mM. SP treatment significantly reduced p62 protein expression levels compared to Ctr siRNA cells, while SP treatment in PPAR−γ siRNA cells showed an increase in p62 protein levels compared to Ctr siRNA cells. (**D**) Densitometric analysis of immunoreactivity to antibodies shown in panel (**B**). *** *p* < 0.001 vs. Ctr siRNA; ** *p* < 0.01 vs. Ctr siRNA; * *p* < 0.05 vs. Ctr siRNA.

Several in vitro and in vivo studies have shown a close correlation between PPAR-γ and short-chain fatty acids (SCFAs), demonstrating that SCFAs possess agonist effects against PPAR-γ [15,43,44]. Here, to support this hypothesis, PPAR-γ gene silencing was performed in U-87 GBM cells which were then treated with SP. First, to verify whether PPAR-γ gene silencing was able to abolish PPAR-γ protein expression, quantitative measurement of PPAR-γ protein expression was performed in GBM cells. Indeed, we confirmed the efficiency of PPAR-γ silencing showing an ~50% decrease in its protein level compared to control cells (Figure 4D,D1). Next, to compare the role exerted by SP on the apoptotic pathway in absence of the PPAR-γ gene, the expression levels of p53, Bax, caspase-3, and Bcl-2 proteins were measured. p53 expression levels were significantly increased in SP-treated cells at the higher concentration of 100 mM, compared to the Ctr siRNA-treated cells. Moreover, the expression of this pro-apoptotic protein was significantly reduced when the PPAR-γ gene was silenced and subsequently treated with 100 mM SP. Similarly, the expression of Bax was also significantly increased when cells were treated with SP alone compared to the Ctr siRNA-treated cells. No significant changes were reported in PPAR-γ-silenced cells that were also treated with SP. Apoptosis was also evaluated through caspase-3 expression level quantification and we observed that there was a significant increase in its expression in SP-treated cells despite no changes reported in PPAR-γ-silenced cells also treated with SP. Conversely, it was observed and confirmed that the anti-apoptotic protein Bcl-2 expression levels were significantly reduced in the SP-treated cells compared to the control cells, while no substantial differences were observed following PPAR-γ silencing and SP treatment (Figure 5A,C). We could appreciate that in absence of the PPAR-γ gene, SP did not show a substantial pro-apoptotic effect, suggesting that SP could upregulate PPAR-γ to influence the apoptosis pathway.

Beyond the apoptotic pathway, we also evaluated proteins involved in the autophagic process like LC3, p62, Beclin-1, and Atg5. We reported that SP-treated cells showed a significant increase in the expression levels of LC3, compared to the Ctr siRNA-treated cells, despite no substantial difference observed in PPAR-γ silenced cells also treated with SP. A similar trend was observed for the Beclin-1 protein which was significantly increased in SP-treated cells. Meanwhile, SP treatment in the absence of the PPAR-γ gene was slightly able to restore the Beclin-1 expression levels. Moreover, the Atg5 expression levels in the SP-treated cells was significantly increased when compared to the control cells, while no significant increase was observed in PPAR-γ silenced cells also treated with SP. In addition, p62 protein expression levels were significantly reduced by SP treatment while PPAR-γ silenced and SP-treated cells showed the p62 protein levels trended towards an increase but not was not statistically significant (Figure 5B,D). Here, these results suggest that the absence of PPAR-γ protein in U-87 GBM cells significantly reduced the effect of SP to modulate the autophagy process.

### 3.2. In Vivo Study

#### 3.2.1. SP Arrested GBM Tumor Growth via PPAR-γ Signaling

**Figure 6:**
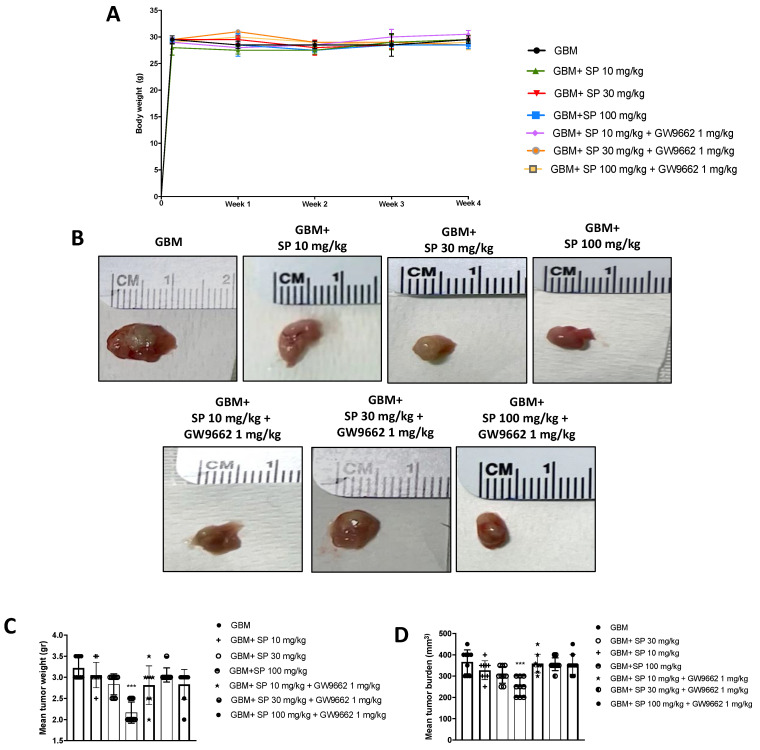
SP treatment reduces GBM tumor growth via PPAR-γ signaling. (**A**) No weight differences were observed between animals treated with SP at a dose of 10, 30, or 100 mg/kg in the GBM group and SP (10, 30 and 100 mg/kg) + GW9661 1 mg/kg groups. (**B**) Representative images of tumor mass appearance from mice subjected to the xenograft model of GBM. (**C**,**D**) A reduction in tumor weight and tumor burden was observed after SP treatments at doses of 30 mg/kg and 100 mg/kg compared to the GBM mice while administration of GW9662 showed no significant differences compared to the GBM mice. *** *p* < 0.001 vs. GBM.

The xenograft model of GBM was used to confirm the data obtained from the in vitro study. The administration of the selective PPAR-γ antagonist GW9662 allowed us to demonstrate that the mechanism of action of SP could be identified as PPAR-γ signaling-dependent. First, no significant changes in the body weight of all mice were reported during the experiment (from day 0 to four weeks). Then, we observed a marked reduction in tumor burden, volume, and weight of tumor samples from SP-treated mice, especially at the dose of 100 mg/kg compared to the GBM group. The lower dose of 30 mg/kg SP did not show significant changes (Figure 6A–D). Moreover, mice treated with GW9662 and SP at doses of 30 and 100 mg/kg, did not show significant reductions in tumor burden, volume, or weight (Figure 6A–D). The tumor burden refers to the size of the tumor mass and it was evaluated by calculating the volume using the caliper measurements, while the weight refers to the weight in grams of the tumor mass. In addition, these parameters were used to assess tumor growth in response to Sodium Propionate treatments.

#### 3.2.2. SP Treatment Resolved GBM Subcutaneous Tumor Mass Features

**Figure 7:**
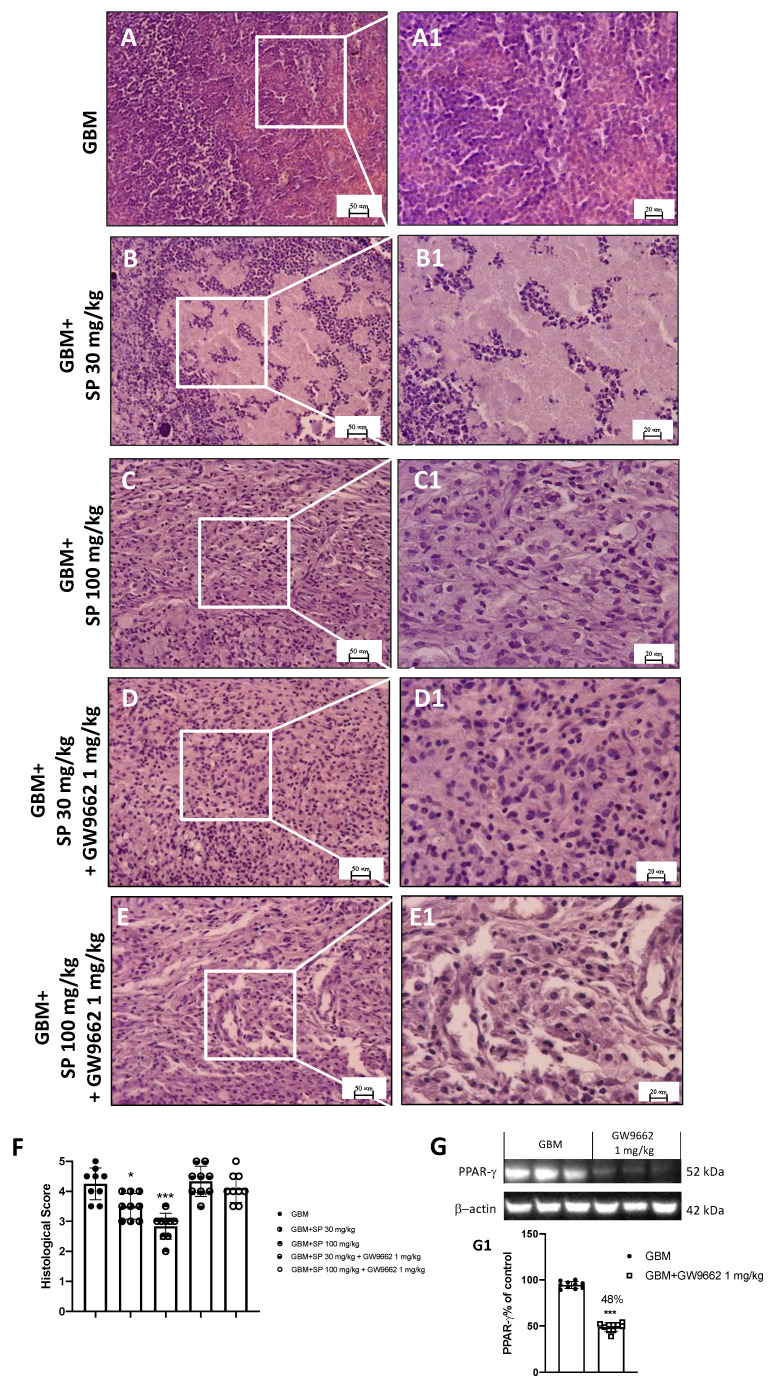
Effect of SP on tumor growth following PPAR-γ antagonist (GW9662) treatment in GBM xenograft model. (**A**) Representative histological image of GBM mice. (**B**,**C**) Treatments with SP at the doses of 30 mg/kg and 100 mg/kg significantly reduced subcutaneous tumor mass as well as neutrophil infiltration compared to GBM mice. (**D**,**E**) Mice treated with GW9662 and SP at the doses of 30 mg/kg and 100 mg/kg showed no significant difference with GBM mice. (**F**) Histologic score. Sections were observed and photographed at 20× and 40× magnification. (**G**) Representative western blot for PPAR-γ in mice treated with GW9662 (1 mg/kg). Densitometric analysis of immunoreactivity to antibody shown in panel (**G1**). * *p* < 0.05 vs. GBM; *** *p* < 0.001 vs. GBM.

Histological analysis revealed that in GBM mice, the subcutaneous tumor mass was characterized by extensive necrotic areas, infiltration of neutrophils, and high-rate tumor/muscle (Figure 7A,A1). Only foci with neutrophils concentrated within and/or immediately adjacent to the tumor cells were considered to define neutrophilic infiltration in tumor sections stained with H&E, while muscle tissue represents the background. Moreover, the tumor necrotic area was characterized by homogeneous groups of dead cells or groups of coalescent cells that form a clot containing nuclear and cytoplasmic debris. Both SP treatments (doses of 30 and 100 mg/kg) were able to attenuate and reduce the pathological features of GBM; in particular, the higher dose of 100 mg/kg significantly reduced neutrophilic infiltration (Figure 7B,B1,C,C1,F). Moreover, when the PPAR-γ antagonist GW9662 was administered in combination with SP, SP action was suppressed by turning off PPAR-γ signaling. Indeed, no significant changes were reported in tissues from GW96621 and SP-treated mice (Figure 7D,D1,E,E1,F). Moreover, we confirmed the efficiency of the PPAR-γ antagonist GW9662 (1 mg/kg) to significantly decrease PPAR-γ levels compared to GBM mice (Figure 7G, see densitometric analysis panel G1).

#### 3.2.3. Proliferation Marker Ki-67 Expression Is Reduced by SP Treatment

**Figure 8:**
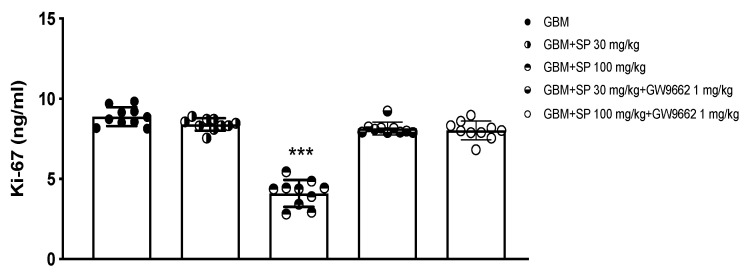
Effect of SP on Ki-67 expression in GBM xenograft model. Ki-67 ELISA assay graph. ***p < 0.001 vs. GBM.

To further study the effect of SP on cell proliferation, the expression of the specific proliferation marker Ki-67 was evaluated using ELISA. Ki-67, a nuclear protein closely associated with cell proliferation, is associated with the progression of neoplastic diseases [45,46]. SP treatment at the higher dose of 100 mg/kg significantly reduced the levels of Ki-67 (Figure 8) compared to the GBM mice (Figure 8) where high levels have been observed despite no significant changes in GBM mice treated with 30 mg/kg SP (Figure 8). Tumor samples from mice treated with the PPAR-γ antagonist GW9662 and SP at the doses of 30 and 100 mg/kg (Figure 8) showed similar expression levels of Ki-67 as GBM mice demonstrating that the reduction of Ki-67 protein expression by SP could be mediated by the PPAR-γ signaling pathway.

#### 3.2.4. SP Treatment Promoted the Apoptosis and Autophagy Pathways in the GBM Tumor Mass through PPAR-γ Signaling

**Figure 9:**
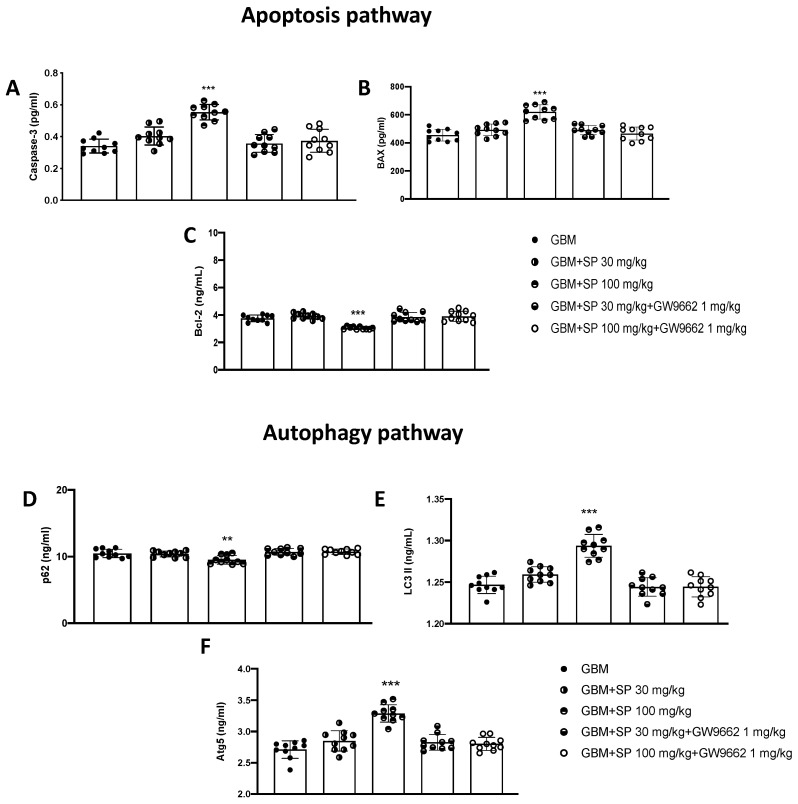
Effect of SP on apoptosis and autophagy markers in GBM tissues. (**A**) Caspase-3 ELISA assay graph. (**B**) Bax ELISA assay graph. (**C**) Bcl-2 ELISA assay graph. (**D**) p62 ELISA assay graph. (**E**) LC3II ELISA assay graph. (**F**) Atg-5 ELISA assay graph. *** *p* < 0.001 vs. GBM; ** *p* < 0.01 vs GBM.

To support the in vitro data, apoptosis and autophagy markers were evaluated by performing ELISA assays. Initially, to evaluate the role of SP on the apoptotic pathway alone and in the presence of the selective PPAR-γ antagonist GW9662, the levels of caspase-3, BAX, and Bcl-2 proteins were measured. The levels of the pro-apoptotic protein caspase3 were significantly increased in animals treated with SP at a dose of 100 mg/kg compared with GBM mice. Furthermore, the expression of this protein was significantly reduced in animals treated with SP (30 mg/kg and 100 mg/kg) + GW9662 (1 mg/kg) (Figure 9A). Similarly, Bax expression was also significantly increased in SP-treated animals at the highest dose of 100 mg/kg compared with GBM mice. Furthermore, no significant changes were reported in SP plus GW9662-treated animals compared to GBM mice (Figure 9B). The opposite trend was observed in the levels of the anti-apoptotic protein Bcl-2 (Figure 9C). Bcl-2 protein levels were significantly reduced in SP 100 mg/kg treated animals compared to GBM mice, while no significant changes were reported in SP and GW9662-treated animals compared to GBM mice. For the evaluation of the autophagic pathway, LC3, p62, and Atg5 proteins were evaluated. p62 expression levels were significantly reduced by SP treatment at a dose of 100 mg/kg compared to GBM mice, while no significant increase was observed in both SP and GW9662-treated mice (Figure 9D). Furthermore, mice treated with SP at the highest dose of 100 mg/kg showed significantly increased LC3 expression levels, compared to GBM mice; on the contrary, no substantial difference was observed in mice treated with SP and GW9662 (Figure 9E). A similar trend was observed for the Atg5 protein which was significantly increased in mice treated with SP at a dose of 100 mg/kg. In contrast, SP and PPAR-γ antagonist treatments did not show any significant increase compared to GBM mice (Figure 9F). From these results it is once again evident that in the presence of a selective PPAR-γ antagonist, SP is unable to modulate the apoptotic and autophagic pathways suggesting that SP could act by upregulating PPAR-γ levels with a molecular mechanism that has yet to be identified.

## 4. Discussion

SCFAs, especially propionate, have been described by several pre-clinical studies as possessing benefits for human health, by reducing inflammatory processes and exerting neuroprotective effects with propionate mentioned as the second strongest activator after butyrate. However, no studies have explored the effect of SP on human GBM cancer cell proliferation and whether it could modulate PPAR-γ signaling. Here, we reported the mechanisms involved in the attenuation of GBM by SP. The two major findings are (i) SP exerts powerful abilities to decrease cell viability, migration, and tumor growth of GBMs by promoting the apoptosis and autophagy pathways, and (ii) SP exerts its effect by PPAR-γ/SCFAs signaling, likely act by upregulating PPAR-γ.

In this study, it was observed that SP treatment selectively de-creased GBM cell viability validating the anti-migration effect observed in U-87 cells treated with SP. Indeed, considering the possibility of GBM cells being affected by SP cytotoxicity, the cell migration test revealed that GBM cells were freely growing to form a confluent monolayer after gap-scratching trace by using their intrinsic migratory ability to invade adjacent tissues. In cells treated with SP, cell migration process seemed to be inhibited, particularly at the higher concentrations of 50 and 100 mM. These preliminary results revealed that SP could link and intercept migratory signals to the cell cytoskeleton of GBM cells and reverse tumor cell spread.

In GBM, aberrant apoptosis promotes tumor progression by overmodulating angio-genesis, migration, and invasive abilities [47]. The effect of SP on the apoptosis pathway was further investigated demonstrating that treatment of U-87 cells with high levels of SP induced cell apoptosis. According to previous reports on SP’s apoptotic effect on colorectal carcinoma cells [48], this effect is mediated by p53 leading to caspase3 activation. In U-87 GBM cells, SP increased apoptosis by decreasing anti-apoptotic Bcl-2 and increasing pro-apoptotic Bax, suggesting that SP acts as a powerful trigger of apoptosis by inducing p53 signaling.

The “double-sword” of autophagy is defined as possessing dual functions, as it can sustain the survival of cancer cells or it can induce cell death [49]. Here, the formation of autophagosome, identified as an increase in LC3II expression levels, indicated that autophagy was activated by SP treatment in U-87 cells. Moreover, the expression levels of p62/SQSTM-1, an autophagy receptor that is degraded when autophagy activation occurs, decreased with increasing concentrations of SP (both doses of 50 and 100 mM). We noted that SP was able to induce cell death through modulation of apoptosis and autophagy pathways that are notably involved in GBM progression. Moreover, the examination of autophagy dynamics in the TME suggested that a correlation between LC3II (lipidated form) and the autophagy-related gene Beclin-1 exists. Loss of Beclin-1 has been reported to promote the progression of astrocytic tumors [50] which explains our observation that Beclin-1 expression levels were low. Thus, we revealed that the SP mechanism to induce autophagy and attenuate GBM progression was mediated by increased expression of Beclin-1 in GBM cells. In addition, the autophagy substrate Atg-5 showed increased expression after SP treatment of GBM cells suggesting that the direct players of the autophagy pathway could be markedly influenced by SP to attenuate GBMs. It is worth noting that enhancing autophagy induced the activation of LC3 and its substrates by SP, which, although still controversial, seems to positively modulate GBM progression [51]. Interestingly, we observed that SP treatments led to an increase in DNA damage by assaying comet tail length and PARP expression in GBM cells. We observed the physical changes of the tail formation and demonstrated that the first stage of DNA dis-aggregation is provided by the short to the long length of the comet tail [52] when cells were treated with SP suggesting that SP could attenuate the DNA damage in the comet tails in U-87 cells.

Among the vast number of different receptors, special attention has been attributed to the role of PPARs in GBM [53] and on their ligands that can inhibit the proliferation of GBM as well as various other tumors [54]. PPARs appear to be activated by a wide array of ligands [55,56,57], including SCFAs such as propionate to reduce mitochondrial dysfunction, oxidative stress, and apoptosis in hepatocytes as well as inhibiting migration of cancer cells [58]. Moreover, PPAR-γ signaling promotes the proliferation of brain metastatic cancer cells [59]. Here, SP treatments upregulated PPAR-γ in GBM cells despite no changes in the other PPARs suggesting that we needed to better define the PPAR-γ/SCFAs interaction and SP’s antitumor activity. Studies carried out in U-87 cells established that GBM cells’ sensitivity to SP can be modulated by PPAR-γ gene silencing as also reflected by the modulation of apoptosis and autophagy pathways. The absence of the PPAR-γ gene poorly stimulating apoptosis because no significant changes were reported when compared to control GBM cells. The increased expression levels of caspase-3, and Bax proteins that are involved in p53 signaling, were associated with SP activity; thus, SP itself possesses powerful effects when the PPAR-γ gene is expressed instead of its absence. The opposite effect was observed for Bcl-2.

Profound interactions between autophagy and apoptosis exist, by mutually reinforcement and inhibition of both pathways in many physiological processes [60]. Thus, considering the interaction PPAR-γ/SCFAs on apoptosis, we further investigated its biological significance in the autophagy pathway. Silencing of the PPAR-γ gene in U-87 cells suppressed the effect mediated by SP, inducing decreasing autophagy mediators resulting in an inhibition of the process. We measured the expression levels of proteins involved in autophagy, such as Beclin-1, LC3, and p62. We observed that SP treatment upregulated Beclin-1, LC3, and Atg-5 when the PPAR-γ gene was not downregulated; the opposite was observed for p62. Therefore, we concluded that SP could increase autophagy activity in GBM cells through the PPAR-γ/SCFAs interaction by further inducing autophagosome formation to deliver unwanted cytoplasmic material to lysosomes for degradation.

Once we demonstrated the molecular mechanism of SP to induce apoptosis and autophagy through PPAR-γ, we next investigated the ability of the PPAR-γ/SCFAs interaction to suppress tumor growth. Malignancy, infiltration of tumor-associated cells, angiogenesis, and tumor growth are common hallmarks of GBM in vivo [61]. Here, SP inhibited tumor growth and weight, as well as tumor burden, compared to the control in a GBM cancer cell xenograft model when orally administered the higher dose of 100 mg/kg SP. The significance of the SP effect against tumorigenesis expanded our understanding of the role of the PPAR-γ/SCFAs. In our study, using the PPAR-γ antagonist GW9662 we re-ported that the co-treatment with SP and GW9662 reduced SP results making us affirm that its action could be directly correlated to the PPAR-γ interaction through upstream actions of SP on the PPAR-γ pathway. Moreover, the pharmacological effects of SP, in counteracting GBM features, revealed that its oral administration attenuated the marked hypercellularity, abnormal matrix, and nuclear atypia compared to the GBM tissue [62]. Accordingly, with these findings, GW9662 treatment in association with SP showed a low ability to resolve GBM histopathological characteristics, consistent with previous studies where PPAR-γ antagonists reversed the protective effects of PPAR-γ agonists [63,64]. Once again, PPAR-γ inhibition attenuated SP’s ability to induce changes in the TME and reversed the GBM tissues’ pathological characteristics.

An additional criterion for assessing the malignancy of GBM is the neuropathological marker Ki67, a nuclear protein expressed in proliferating cells. In GBM tissues, Ki-67 expression was upregulated, consistent with tumor grade and previous studies [65,66]. In addition, SP reduced GBM invasion in U-87 as demonstrated by a decreased invasion of tumor cells into the adjacent parenchyma. Moreover, considering the low expression of the Ki67 marker in SP-treated mice, the next results demonstrated a higher proliferation index (Ki67 immunopositivity) of GBM tissue from mice co-treated with GW9662 and SP that was similar to that of the control. This clearly shows that SP decreases tumor proliferation through PPAR-γ resulting in the loss of tumorigenicity due to the PPAR-γ/SCFAs interaction.

## 5. Conclusions

In conclusion, our results provide a line of evidence indicating that SP promotes the apoptosis and autophagy pathways through a PPAR-γ-dependent mechanism, inducing the slowing of tumor progression and the cell cycle in GBM. Therefore, the PPAR-γ/SCFAs interaction is a potential candidate for the management of GBM because of SP’s ability to counteract tumorigenesis.

## Data Availability

The data presented in this study are available on request from the corresponding author.
